# Spreadability for Quantum Stochastic Processes, with an Application to Boolean Commutation Relations

**DOI:** 10.3390/e22050532

**Published:** 2020-05-08

**Authors:** Vitonofrio Crismale, Francesco Fidaleo, Maria Elena Griseta

**Affiliations:** 1 Dipartimento di Matematica, Università degli studi di Bari, Via E. Orabona, 4, 70125 Bari, Italy; vitonofrio.crismale@uniba.it (V.C.); maria.griseta@uniba.it (M.E.G.); 2 Dipartimento di Matematica, Università degli studi di Roma Tor Vergata, Via della Ricerca Scientifica 1, 00133 Roma, Italy

**Keywords:** noncommutative probability, noncommutative dynamical systems, spreadable stochastic processes, states, 60G09, 05A99, 16S35, 46L53, 46L55, 46L30

## Abstract

In order to manage spreadability for quantum stochastic processes, we study in detail the structure of the involved monoids acting on the index-set of all integers Z, that is that generated by left and right hand-side partial shifts, the monoid of all strictly increasing maps whose range has finite complement, and finally the collection of all strictly increasing maps of Z. We show that such three monoids are strictly ordered, and the second-named one is the semidirect product between the first and the action of Z generated by the one-step shift. Even if the definition of a spreadable stochastic process is provided in terms of the invariance of the finite joint distributions under the natural action of the last monoid on the indices, we see that spreadability can be directly stated in terms of invariance with respect to the action of the first monoid. Concerning the stochastic processes involving the concrete boolean $C^{*}$-algebra generated by the annihilators acting on the boolean Fock space (i.e., the concrete $C^{*}$-algebra satisfying the boolean commutation relations), we study their spreadability directly in terms of the invariance under the monoid generated by all strictly increasing maps whose range has finite complement because, for this case, such an investigation appears more direct and manageable. Finally, we present the version of the Ryll–Nardzewski theorem for the boolean case, establishing that spreadable, exchangeable and stationary stochastic processes coincide, and describing their common structure.

## 1. Introduction

Stochastic processes invariant under distributional symmetries have been intensively studied in classical probability theory, and their natural applications to statistical mechanics and other applied fields deeply encouraged this investigation. The reader is referred to [1] for an exhaustive account on the matter. It was then natural to address the systematic investigation of the theory of stochastic processes to the quantum setting, which indeed started with the seminal paper [2].

Families of random variables which are not necessarily commutative provide a general framework to realise de Finetti-type theorems, and therefore to classify stochastic processes whose finite joint distributions are independent of the action of some algebraic structures. Potential applications to quantum information theory and quantum statistical mechanics promoted a huge amount of results in this subject in recent years. For an account which is far to be complete, we refer the reader to [3,4,5,6,7], and the references cited therein.

Among the most common distributional symmetries, we mention spreadability, exchangeability and stationarity. By definition, exchangeable stochastic processes are automatically spreadable. For commutative random variables, the converse also holds. The equivalence between spreadability and exchangeability is indeed the Ryll–Nardzewki theorem (cf. [8]), nowadays celebrated as a part of the so-called extended de Finetti theorem. This statement is not generally true in the non commutative setting (e.g., [9]), but there are prominent examples of quantum stochastic processes for which it still holds. One of them is the boolean case, as we are going to show in these notes.

Recently, in quantum probability it has been established a one-to-one correspondence between unitarily equivalent classes of noncommutative stochastic processes on the index-set *J*, for the sample $C^{*}$-algebra A, and states on the free product $C^{*}$-algebra ${*}_{J}A$. This entails that exchangeable or stationary (in the case J=Z) stochastic processes are uniquely determined by symmetric or shift invariant states on ${*}_{J}A$, respectively. The same holds on concrete $C^{*}$-algebras, seen as the quotient of the free product $C^{*}$-algebra, by means of the universal property of ${*}_{J}A$. The reader is referred to [4,10] for more details about this conceptual point.

Using these results, in [5] spreadability was investigated for stochastic processes arising from the so-called monotone commutation relations. There, it was shown that the monoid generated by the right and left hand-side partial shifts acts on the monotone $C^{*}$-algebra by unital *-endomorphisms, and spreadable stochastic processes, or equivalently spreading invariant states, were also classified.

After studying the algebraic structures involved in spreadability, here we develop the analysis of such a distributional symmetry for processes belonging to the concrete $C^{*}$-algebra arising from boolean commutation relations. One of the main interest in this research field is motivated by the physical application in quantum optics of such boolean stochastic processes, as pointed out in [11].

We mention that the structure of the (concrete) boolean $C^{*}$-algebra was investigated in [10,12], whereas the ergodic properties of invariant states (see e.g., [13]) for the monotone vs. boolean $C^{*}$-algebras, with their similarities and differences, are described in [14], Section 5.

The paper is organised as follows. After recalling in Section 2 some features on $C^{*}$-dynamical systems and quantum stochastic processes, in Section 3 we present the distributional symmetries managed in the notes, and some of the basic relations among them as well. Although the definition of a spreadable stochastic process is provided in terms of the invariance of the finite joint distributions under the natural action of the monoid of strictly increasing maps on Z, here we show that spreadability can be directly stated in terms of invariance with respect to the action of the monoid, denoted by $I_{Z}$, generated by left and right hand-side partial shifts on the integers.

In Section 4, we introduce a further monoid involved in our investigation, that is the one generated by the strictly increasing maps on Z whose range has finite complement. It is denoted by $J_{Z}$, and we see that it is properly included in the monoid of strictly increasing maps, and strictly contains $I_{Z}$. In addition, it provides another structure to study spreadability, as we show that spreading invariant states are exactly those invariant under the action of $J_{Z}$. This statement appears interesting in our successive investigation about boolean stochastic processes, since $J_{Z}$ offers a more flexible analysis in that case. The intimate relation between $J_{Z}$ and $I_{Z}$ is pointed out in Proposition 4, which is the main result of the section. There, we prove that $J_{Z}$ is the semi-direct product of $I_{Z}$ by the action of Z generated by the conjugation by the one-step shift. Such a result of combinatory nature, has a natural self-containing interest deserving of possible applications in other fields of mathematics where the monoids of increasing maps can play a role.

In Section 5, it is seen that $J_{Z}$ acts by unital *-endomorphisms on the concrete boolean $C^{*}$-algebra. In particular, this result is preliminary achieved on the unital *-algebra of finite rank operators on the boolean Fock space. Then, it is extended to its closure in the uniform topology (i.e., the $C^{*}$-algebra of the compact linear operators), which indeed coincides with the (concrete) boolean $C^{*}$-algebra, and the action is completely described also in this case. This result is also aimed to yield the structure of boolean spreading invariant states, given in the last part of the notes. Indeed, we get the suitable version of the Ryll–Nardzewki theorem in our setting, finding that the states invariant under the aforementioned action are exactly the symmetric, or equivalently shift invariant positive normalised functionals, whose common structure was obtained in [15] and [10], respectively.

In the final section, we briefly summarise some open problems for further investigations.

## 2. Preliminaries

The present section is devoted to collect some features and properties useful in the forthcoming part of the notes. To shorten the notations, we indifferently denote by N the set of all natural numbers, with or without 0, if this causes no confusion. In addition, we put

Z⊃[k,l]:={j∈Z∣k≤j≤l}.

In our setting, the triplet (A,M,Γ) is said a $C^{*}$-dynamical system if A is a $C^{*}$-algebra with unit $1\;\;I_{A}=:1\;\;I$, *M* is a monoid, and finally Γ is a representation $g\in M\mapsto \Gamma_{g}$ of *M* by completely positive identity preserving (i.e., unital) maps of A.

In some cases, *M* is replaced by a group *G*, and in the $C^{*}$-dynamical system (A,G,α)α is indeed a representation of *G* into the group of the *-automorphisms Aut(A) of A. In the latter case, one speaks of reversible dynamics, whereas dissipative dynamics appears in absence of bijections, see, e.g., [16].

By S(A) we denote the convex of the states on A, that is the positive normalised linear functionals on A. S(A) is weakly *-compact as A is unital.

Let φ∈S(A) be invariant under the action of each element of *M*, i.e., $\varphi \circ \Gamma_{g}=\varphi$, g∈M, and consider the Gelfand–Naimark–Segal (GNS for short) representation $\left(H_{\varphi },\pi_{\varphi },\xi_{\varphi }\right)$. Then there exists a unique contraction $V_{\varphi ,g}\in B\left(H_{\varphi }\right)$ such that $V_{\varphi ,g}\xi_{\varphi }=\xi_{\varphi }$ and

$$V_{\varphi ,g}\pi_{\varphi }\left(a\right)\xi_{\varphi }=\pi_{\varphi }\left(\Gamma_{g}\left(a\right)\right)\xi_{\varphi }\;,\;a\in A\;,$$

see, e.g., [17], Lemma 2.1. The quadruple $\left(H_{\varphi },\pi_{\varphi },V_{\varphi ,g},\xi_{\varphi }\right)$ is called the covariant GNS representation associated to the invariant state φ. If the $\Gamma_{g}$ are multiplicative, the $V_{\varphi ,g}$ are isometries. If in addition the $\Gamma_{g}$ are invertible, then the $V_{\varphi ,g}$ are unitaries.

The convex, compact in the *-weak topolgy, subset of all invariant states is

$$S_{M}\left(A\right):=\left\{\varphi \in S\left(A\right)|\varphi \circ \Gamma_{g}=\varphi \;,\;\;g\in M\right\}\;.$$

The set of the extremal invariant states (i.e., the extreme boundary) is denoted by $E_{M}\left(A\right):=\partial S_{M}\left(A\right)$. Those are, by definition, nothing else than the ergodic states under the action Γ of *M*.

Among the groups we deal with, we mention that consisting of all permutations $P_{J}$ of an arbitrary index-set *J*, leaving fixed all elements but finitely many. It is given by

$$P_{J}:=⋃\left\{P_{\Lambda }|\Lambda \;\mathrm{finite}\;\mathrm{subset}\;\mathrm{of}\;J\right\},$$

where $P_{\Lambda }$ is the symmetric group associated to the finite set Λ. If *J* is the linearly ordered Z, we also mention the group generated by the one-step shift τ(i):=i+1 of the integers Z, which is canonically identified with Z itself.

Recall that, for a given arbitrary set *J* and unital $C^{*}$-algebras ${\left\{A_{j}\right\}}_{j\in J}$, their unital free product $C^{*}$-algebra ${*}_{j\in J}A_{j}$ (cf. [18]) is the unique unital $C^{*}$-algebra, together with unital monomorphisms $i_{j}:A_{j}\to {*}_{j\in J}A_{j}$ such that for any unital $C^{*}$-algebra B and unital morphisms $\Phi_{j}:A_{j}\to B$, there exists a unique unital homomorphism $\Phi :{*}_{j\in J}A_{j}\to B$ making commutative the following diagram

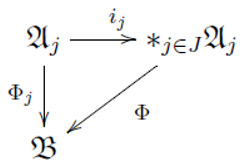


Here, we consider unital free product $C^{*}$-algebras based on a single unital $C^{*}$-algebra A, the algebra of the samples, called the free product $C^{*}$-algebra, and denoted simply as ${*}_{J}A$. We refer the reader to [4,10,19] for further details.

The central aspect in the theory of stochastic processes is to construct a process based on the sample algebra A and the index-set *J*, starting from the knowledge of the collection of its finite dimensional distributions. In the abelian case the suitable conditions are summarised in the Kolmogorov Reconstruction Theorem, whereas the quantum generalisation is provided by the GNS construction.

To be more precise, fix n=1,2,…, $\{j_{1},j_{2},\ldots ,j_{n}\}\subseteq J$ with contiguous different indices, and $\{A_{1},A_{2}\ldots ,A_{n}\}\subseteq A$. The finite joint distributions are the values $p_{j_{1},j_{2},\ldots ,j_{n}}\left(A_{1},A_{2},\ldots ,A_{n}\right)$ which arise from multilinear functionals ${\left\{p_{j_{1},j_{2},\ldots ,j_{n}}\right\}}_{j_{1},j_{2},\ldots ,j_{n}\in J}$ on A. They satisfy some natural positivity and consistency conditions given by

$$\begin{array}{cc} (i)\;\;\;\;\;\; & p_{j_{n},\ldots ,j_{2},j_{1},j_{2},\ldots ,j_{n}}\left(A_{n}^{*},\ldots ,A_{1}^{*}A_{1},\ldots ,A_{n}\right)\geq 0\;\;\left(positivity\right)\\ (ii)\;\;\;\;\; & p_{j_{1},\ldots ,j_{k-1},j_{k},j_{k-1},\ldots ,j_{n}}\left(A_{1},\ldots ,A_{k-1},1\;\;I,A_{k+1},\ldots ,A_{n}\right)\\ = & p_{j_{1},\ldots ,j_{k-1},j_{k+1},\ldots ,j_{n}}\left(A_{1},\ldots ,A_{k-1},A_{k+1},\ldots ,A_{n}\right)\;\;\left(consistency\right). \end{array}$$

The classical case is characterised by A=C(X), *X* being a (locally) compact space, and

(1) $$p_{j_{g(1)},j_{g(2)},\ldots ,j_{g(n)}}\left(A_{g(1)},A_{g(2)},\ldots ,A_{g(n)}\right)=p_{j_{1},j_{2},\ldots ,j_{n}}\left(A_{1},A_{2},\ldots ,A_{n}\right)$$

for $g\in P_{\left[1,n\right]}$, $A_{1},A_{2},\ldots ,A_{n}\in A$, and n=1,2,….

The above properties indeed reduce to the Kolmogorov requests, and Equation (1) gives that a classical stochastic process is uniquely determined, up to equivalence, by the finite joint distributions $p_{j_{1},j_{2},\ldots ,j_{n}}$ such that the sets of indices $\{j_{1},j_{2},\ldots ,j_{n}\}\subseteq J$ are all different, and independent of their order.

Thus, the Kolmogorov theorem allows to construct a probability measure μ on the Tikhonoff product $\prod_{J}X$ of *J* copies of *X*. In the quantum setting, the aforementioned properties permit to perform the GNS representation (defined up to unitary equivalence), and so give rise to general stochastic processes as defined in the forthcoming lines.

By taking into account the previous considerations, we can assume as starting point (i.e., by definition) that the process under consideration is directly realised on a Hilbert space.

A (realisation of a) possibly quantum, stochastic process, labelled by the index-set *J* and determined up to unitary equivalence is, in our language, a quadruple $\left(A,H,{\left\{\iota_{j}\right\}}_{j\in J},\xi \right)$, where A is a $C^{*}$-algebra, H is an Hilbert space, the maps $\iota_{j}$ are *-homomorphisms of A in B(H), and ξ∈H is a unit vector, cyclic for the von Neumann algebra $M:={⋁}_{j\in J}\iota_{j}\left(A\right)$ naturally acting on H.

In [10], Theorem 3.4, it was proved that states on ${*}_{J}A$ uniquely correspond to quantum stochastic processes. More in detail, one sees that the quadruple $\left(A,H,{\left\{\iota_{j}\right\}}_{j\in J},\xi \right)$ determines a unique state $\varphi \in S\left({*}_{J}A\right)$, and a representation π of ${*}_{J}A$ on the Hilbert space H, such that (π,H,ξ) is the GNS representation of the state φ.

Conversely, each state $\varphi \in S\left({*}_{J}A\right)$ defines a unique stochastic process, just by looking at its GNS representation uniquely determined up to unitary equivalence.

For more details and proofs, the interested reader is referred to the above mentioned paper, and [4] as well.

## 3. Stochastic Processes and Their Symmetries

In the present section, we investigate some natural invariance properties for the stochastic processes. Among those, we will deal with the so-called stationarity, spreadability and exchangeability. To simplify the matter, we suppose that J=Z in order to compare the above three mentioned symmetries.

We consider the set $Z^{Z}$ of all maps f:Z→Z. It provides a monoid $\left(Z^{Z},\circ ,{\mathrm{id}}_{Z}\right)$, where the product is the composition “∘” between maps and the unit $e_{Z^{Z}}$ is the identity-map ${\mathrm{id}}_{Z}$ of Z.

The following two sub-monoids of $\left(Z^{Z},\circ ,{\mathrm{id}}_{Z}\right)$ are of interest for our analysis. The first one

$$L_{Z}:=\left\{f:Z\to Z|k<l\Rightarrow f(k)<f(l)\right\}$$

is given by all the strictly increasing maps of Z, or equivalently maps which determine all subsequences of Z. Obviously, if $f,g\in L_{Z}$ then their composition $f\circ g\in L_{Z}$, and therefore $L_{Z}$ is endowed with a structure making it a monoid $\left(L_{Z},\circ ,{\mathrm{id}}_{Z}\right)$.

The second one is the monoid generated by all partial shifts on Z. Namely, the *h*-right hand-side partial shift, h∈Z, is the one-to-one map $\theta_{h}:Z\to Z$ such that

$$\theta_{h}\left(k\right):=\left\{\begin{array}{cc} k & \mathrm{if}\;\;k<h\;,\\ k+1 & \mathrm{if}\;\;k\geq h\;. \end{array}\right.$$

Analogously, the *h*-left hand-side partial shift, h∈Z, is the one-to-one map $\psi_{h}:Z\to Z$ such that

$$\psi_{h}\left(k\right):=\left\{\begin{array}{cc} k & \mathrm{if}\;\;k>h\;,\\ k-1 & \mathrm{if}\;\;k\leq h\;. \end{array}\right.$$

We note that $\left\{\theta_{h}\left(k\right),\psi_{h}\left(k\right)|k\in Z\right\}\subseteq L_{Z}$.

Let us denote by $\left(I_{Z},\circ ,{\mathrm{id}}_{Z}\right)$ the sub-monoid of $\left(L_{Z},\circ ,{\mathrm{id}}_{Z}\right)$ generated by all forward and backward partial shifts ${\left\{\theta_{h}\right\}}_{h\in Z}$, and ${\left\{\psi_{h}\right\}}_{h\in Z}$. We will see later that $\left(I_{Z},\circ ,{\mathrm{id}}_{Z}\right)⊊\left(L_{Z},\circ ,{\mathrm{id}}_{Z}\right)$.

From now on, we drop the composition symbol simply by writing that as a product: f∘g≡fg. Thus, we indicate with $f^{n}$ the *n*-fold composition of *f* with itself. If $f\in Z^{Z}$ is invertible, we put $f^{-n}:=\underset{n-\mathrm{times}}{\underset{︸}{f^{-1}\circ \cdots \circ f^{-1}}}\;,$ and therefore $f^{n}$ is defined for all n∈Z when it is meaningful.

We often also write the relative monoids without pointing out the composition and the unit. For example, $\left(I_{Z},\circ ,{\mathrm{id}}_{Z}\right)$ and $\left(L_{Z},\circ ,{\mathrm{id}}_{Z}\right)$ will be denoted simply as $I_{Z}$ and $L_{Z}$, respectively.

**Remark** **1.** *The powers of the one-step shift τ and its inverse $\tau^{-1}$ act in a natural way on $Z^{Z}$ by conjugacy: for $f\in Z^{Z}$,*

(2) $$\eta_{m}\left(f\right)\left(l\right):=\left(\tau^{m}f\tau^{-m}\right)\left(l\right)=f\left(l-m\right)+m\;,\;\;\;m,l\in Z\;.$$

*Therefore, $\eta_{m}\in \mathrm{Aut}\left(Z^{Z}\right)$ for any m∈Z.*

We report, without the proof, the following useful results (cf. [10], Lemma 2.2, and [5], Proposition 2.1) in order to manage the symmetries of stochastic processes which will be introduced below.

**Proposition** **1.** *The following holds true for a finite interval [k,l]⊂Z.*

*(i)*  *There exists a cycle $\sigma_{k,l}\in P_{Z}$ such that $\tau \left(\left[k,l\right]\right)=\sigma_{k,l}\left(\left[k,l\right]\right)$.*
*(ii)*  *For each $f\in L_{Z}$ there exists $r_{k,l;f}\in I_{Z}$ such that $f\left(\left[k,l\right]\right)=r_{k,l;f}\left(\left[k,l\right]\right)$.*

By universality, the groups Z and $P_{Z}$ act in a natural way as *-automorphisms on the free product $C^{*}$-algebra ${*}_{Z}A$ by shifting and permuting the indices of the generators, respectively. Moreover, it is possible to see (cf. [5], Section 4) that there is an action by *-endomorphisms of both the monoids $I_{Z}$ and $L_{Z}$ on ${*}_{Z}A$.

Denoting by $\left({*}_{Z}A,\tau ,Z\right)$, $\left({*}_{Z}A,\pi ,P_{Z}\right)$, $\left({*}_{Z}A,\Gamma ,I_{Z}\right)$, and $\left({*}_{Z}A,\Gamma ,L_{Z}\right)$ the corresponding dynamical systems, we have the following immediate consequences of Proposition 1.

**Corollary** **1.** *The following assertions hold true:*

*(i)*  *$S_{P_{Z}}\left({*}_{Z}A\right)\subseteq S_{Z}\left({*}_{Z}A\right)$.*
*(ii)*  *$S_{L_{Z}}\left({*}_{Z}A\right)=S_{I_{Z}}\left({*}_{Z}A\right)$.*

**Proof.**  Taking into account the proof of Proposition 2.1 in [4], (i) and the relation $S_{I_{Z}}\left({*}_{Z}A\right)\subseteq S_{L_{Z}}\left({*}_{Z}A\right)$ follow directly from (i) and (ii) of Proposition 1, respectively. Since $I_{Z}\subseteq L_{Z}$, and therefore $S_{L_{Z}}\left({*}_{Z}A\right)\subseteq S_{I_{Z}}\left({*}_{Z}A\right)$, (ii) holds true as well. □

**Definition** **1.** *For the $C^{*}$-algebra A, n∈N, $j_{1},\ldots j_{n}\in Z$, $A_{1},\ldots A_{n}\in A$, the stochastic process $\left(A,H,{\left\{\iota_{j}\right\}}_{j\in Z},\xi \right)$ is said to be*

**-**  *stationary if*
  $$\langle \iota_{j_{1}}\left(A_{1}\right)\cdots \iota_{j_{n}}\left(A_{n}\right)\xi ,\xi \rangle =\langle \iota_{j_{1}+1}\left(A_{1}\right)\cdots \iota_{j_{n}+1}\left(A_{n}\right)\xi ,\xi \rangle \;;$$
**-**  *exchangeable if for each $g\in P_{Z}$,*
  $$\langle \iota_{j_{1}}\left(A_{1}\right)\cdots \iota_{j_{n}}\left(A_{n}\right)\xi ,\xi \rangle =\langle \iota_{g(j_{1})}\left(A_{1}\right)\cdots \iota_{g(j_{n})}\left(A_{n}\right)\xi ,\xi \rangle \;;$$
**-**  *spreadable if for each $g\in L_{Z}$,*
  $$\langle \iota_{j_{1}}\left(A_{1}\right)\cdots \iota_{j_{n}}\left(A_{n}\right)\xi ,\xi \rangle =\langle \iota_{g(j_{1})}\left(A_{1}\right)\cdots \iota_{g(j_{n})}\left(A_{n}\right)\xi ,\xi \rangle \;.$$

By the above mentioned equivalence between stochastic processes for the sample algebra A on the index-set Z, and states on ${*}_{Z}A$, Corollary 1 leads to:

**-**  If a process is exchangeable, then it is stationary;
**-**  A process is spreadable if and only if it is invariant under the natural action of the monoid $I_{Z}$.

Therefore, we can indifferently define spreadability as the invariance under the action of $L_{Z}$, or equivalently the invariance under its sub-monoid $I_{Z}$. We will see later that, for particular models, spreadability can be conveniently investigated by a suitable monoid included between $L_{Z}$ and $I_{Z}$.

## 4. Monoids of Increasing Maps

In order to manage spreadability, it appears useful to define and study the structure of further sub-monoids of $L_{Z}$.

Let us denote by $\left(D_{Z},\circ ,{\mathrm{id}}_{Z}\right)$ and $\left(E_{Z},\circ ,{\mathrm{id}}_{Z}\right)$ the sub-monoids of $\left(L_{Z},\circ ,{\mathrm{id}}_{Z}\right)$ generated by all forward and backward partial shifts ${\left\{\theta_{h}\right\}}_{h\in Z}$ and ${\left\{\psi_{h}\right\}}_{h\in Z}$, respectively. In addition, let us take

$$J_{Z}:=\left\{f\in L_{Z}:\left|Z\setminus f\left(Z\right)\right|<+\infty \right\}\;.$$

$\left(J_{Z},\circ ,{\mathrm{id}}_{Z}\right)$ is also a sub-monoid of $\left(L_{Z},\circ ,{\mathrm{id}}_{Z}\right)$. As usual, we denote such monoids simply by $D_{Z}$, $E_{Z}$ and $J_{Z}$, respectively. We note that all such monoids are sub-monoids of $Z^{Z}$.

**Remark** **2.** *We have $D_{Z}\cap E_{Z}=\left\{{\mathrm{id}}_{Z}\right\}$.*

**Proof.**  For $f\in D\setminus \{{\mathrm{id}}_{Z}\}$, there exists $j_{f}\in Z$ such that ${f\lceil }_{\left(-\infty ,j_{f}\right]}={\mathrm{id}}_{Z}\lceil_{\left(-\infty ,j_{f}\right]}$. On the other hand, if $g\in E\setminus \{{\mathrm{id}}_{Z}\}$ then there exists $k_{g}\in Z$ such that ${g\lceil }_{\left(-\infty ,m\right]}\neq {\mathrm{id}}_{Z}\lceil_{\left(-\infty ,m\right]}$ for each $m\leq k_{g}$. This concludes the proof. □

For $f\in L_{Z}$, we provide the following notation

$$\delta_{f}:=Z\setminus f\left(Z\right)\;.$$

We have:

**-**  $f\in J_{Z}\Leftrightarrow \delta_{f}<+\infty$,
**-**  $f=\tau^{n}$ for some $n\in Z\Leftrightarrow \delta_{f}=\emptyset$.

**Remark** **3.** *Let us take $f,g\in J_{Z}$. Then one has*

(3) $$\delta_{f}\cup f\left(\delta_{g}\right)=\delta_{fg}\;.$$

**Proof.**  Fix $f,g\in J_{Z}$. Since *f* is one-to-one, it follows

$$f\left(\delta_{g}\right)=f\left(Z\right)\setminus f\left(g\left(Z\right)\right)\;.$$

This gives

$$\delta_{f}\cup f\left(\delta_{g}\right)=Z\setminus f\left(g\left(Z\right)\right)=\delta_{fg}\;.$$

 □

Here, we investigate the relations among the above introduced structures. To our goal, we start with the following technical

**Lemma** **1.** *Let $f\in I_{Z}\setminus \left\{{\mathrm{id}}_{Z}\right\}$. Namely $f=\nu_{h_{1}}\nu_{h_{2}}\cdots \nu_{h_{n}}$, where $\nu_{h_{i}}=\theta_{h_{i}}$, or $\nu_{h_{i}}=\psi_{h_{i}}$ for some $h_{i}\in Z$, i=1,…,n, and n∈N. Then $|\delta_{f}|=n$.*

**Proof.**  We start by noticing that, if $f=\nu_{h}$, one finds $\delta_{f}=\left\{h\right\}$. Now we proceed by induction.

Indeed, suppose that for $g=\nu_{h_{1}}\cdots \nu_{h_{n}}$, where $h_{i}\in Z$, i=1,…,n and n∈N, one has $|\delta_{g}|=n$. After taking $f:=\nu_{h}g$, h∈Z, we prove $|\delta_{f}|=n+1$. We reduce the matter to $\nu_{h}=\theta_{h}$, the other case being similar. Here, since $\delta_{g}=\left\{m_{1},\ldots ,m_{n}\right\}$ for some $m_{1}<\cdots <m_{n}$, one finds for $m_{j-1}<h\leq m_{j}$ and 2≤j≤n,

$$\delta_{f}=\left\{m_{1},\ldots ,m_{j-1},h,m_{j}+1,\ldots ,m_{n}+1\right\}\;,$$

whereas $h\leq m_{1}$ gives

$$\delta_{f}=\left\{h,m_{1}+1,\ldots ,m_{n}+1\right\}\;,$$

and finally

$$\delta_{f}=\left\{m,\ldots ,m_{n},h\right\}$$

when $m_{n}<h$. □

**Remark** **4.** *Corollary 1 and Lemma 1 lead to*

*(iii)*  *$S_{L_{Z}}\left({*}_{Z}A\right)=S_{J_{Z}}\left({*}_{Z}A\right)=S_{I_{Z}}\left({*}_{Z}A\right)$.*

Therefore, spreadability for a stochastic process can be equivalently defined in terms of invariance w.r.t. the action of the monoid $J_{Z}$. This turns out to be more convenient in the case of (concrete) boolean processes, as we will see later.

Lemma 1 allows to prove that some of the monoids introduced above are strictly ordered with respect to set inclusion.

**Proposition** **2.** *Under the notations above, one finds*

$$I_{Z}⊊J_{Z}⊊L_{Z}$$

**Proof.**  Lemma 1 gives $I_{Z}\subseteq J_{Z}$ and, by definition, $J_{Z}\subseteq L_{Z}$. Moreover, the strictly increasing map

s:n∈Z↦s(n):=2n∈Z

does not belong to $J_{Z}$, as $|\delta_{s}|=\infty$. In addition, the one-step shift τ clearly belongs to $J_{Z}$. However, since $\delta_{\tau }=\emptyset$, $\tau \notin I_{Z}$ by Lemma 1. □

The structure of $J_{Z}$ is strictly related to that of $I_{Z}$. For this purpose, we recall the notion of semi-direct product of two monoids *M* and *N*, generalising the analogous notion for groups.

Indeed, fix a monoid *M* acting by morphisms

$$M∋m\mapsto \eta_{m}\in \mathrm{Mor}\left(N\right)$$

on a second monoid *N*. The semi-direct product $M{\;}_{\eta }\;\;⋉N$ is defined as follows. As a set, $M{\;}_{\eta }\;\;⋉N:=M\times N$, whereas a binary operation is given by

(4) $$\left(m_{1},n_{1}\right)\left(m_{2},n_{2}\right):=\left(m_{1}m_{2},n_{1}\eta_{m_{1}}\left(n_{2}\right)\right)\;.$$

It is easy to check that M×N, equipped with the multiplicative law in Equation (4) defines a monoid whose unit is $e_{M\;{\;}_{\eta }\;⋉N}=\left(e_{M},e_{N}\right)$.

**Proposition** **3.** *The group Z acts on the monoids $L_{Z}$, $J_{Z}$, $D_{Z}$, $E_{Z}$ and $I_{Z}$, through the powers of one-step shift τ and its inverse $\tau^{-1}$.*

**Proof.**  Fix m∈Z. It is immediate to see that the map $\eta_{m}$ defined in Equation (2) realises an automorphism when restricted to $L_{Z}$, that is Z acts on $L_{Z}$. Since $|\delta_{\eta_{m}\left(f\right)}\left|=\right|\delta_{f}|$, $f\in J_{Z}$, one has that Z acts by restriction on $J_{Z}$. By [5], Section 2.3, Z acts by restriction, separately on $D_{Z}$ and $E_{Z}$, and therefore also on $I_{Z}$ which is generated by the latter monoids. □

Here, there is the main result of the present section.

**Proposition** **4.** *Under the above notations, one has*

$$J_{Z}=Z{\;}_{\eta }\;\;⋉I_{Z}=Z{\;}_{\eta }\;\;⋉D_{Z}=Z{\;}_{\eta }\;\;⋉E_{Z}\;.$$

**Proof.**  We first note that, for k,l∈Z,

(5) $$\begin{array}{l} \eta_{l}\left(\theta_{k}\right)=\tau^{l}\theta_{k}\tau^{-l}=\theta_{l+k}\;,\\ \eta_{l}\left(\psi_{k}\right)=\tau^{l}\psi_{k}\tau^{-l}=\psi_{l+k}\;. \end{array}$$

If $f\in J_{Z}$, we show that *f* is uniquely decomposed as $f=h\tau^{m}$, for $h\in D_{Z}$ and m∈Z.

In order to prove the claim, we preliminary observe that for any $f\in J_{Z}$ there exist uniquely determined k,m∈Z (depending on *f*) such that

$${f\lceil }_{\left(-\infty ,k\right]}=\tau^{m}\lceil_{\left(-\infty ,k\right]}\;.$$

If $g:=\tau^{-m}f$, it results $g\in J_{Z}$. Hence, $\delta_{g}=\left\{i_{1}\;\ldots ,i_{n}\right\}$ for some integers $i_{1}<\cdots <i_{n}$, and n∈N. Note that $i_{1}=k+1$, as for the strictly increasing *g* one finds ${g\lceil }_{\left(-\infty ,k\right]}={\mathrm{id}}_{Z}\lceil_{\left(-\infty ,k\right]}$.

For each j=1,…,n−1, one can have either $i_{j+1}=i_{j}+1$ or $i_{j+1}>i_{j}+1$. Thus, one defines $A:=\{j=1,\ldots ,n-1|i_{j+1}>i_{j}+1\}$, and denotes s:=|A| where, as usual, s=0 if A=∅.

In the case s=0, the aforementioned decomposition is achieved, since one finds

$$f=\tau^{m}\theta_{k+1}^{n}=\theta_{k+1+m}^{n}\tau^{m}\;,$$

the last equality following from Equation (5).

If instead *A* is non-void, we write $A=\{j_{1},\ldots ,j_{s}\}$ for some $j_{1}<\cdots <j_{s}$, and obtain

$$\left[i_{1},i_{n}\right]=\overset{s+1}{\underset{j=1}{⋃}}\delta^{\left(j\right)}\cup \overset{s}{\underset{j=1}{⋃}}\lambda^{\left(j\right)}\;,$$

where

$$\begin{array}{cc} \; & \delta^{\left(1\right)}:=\left\{i_{1},\ldots ,i_{j_{1}}\right\}\;,\\ \; & \delta^{\left(s+1\right)}:=\left\{i_{j_{s}+1},\ldots ,i_{n}\right\}\;,\\ \; & \delta^{\left(l\right)}:=\left\{i_{j_{l-1}+1},\ldots ,i_{j_{l}}\right\}\;,\;l=2,\ldots ,s\;,\\ \; & \lambda^{\left(l\right)}:=\left\{i_{j_{l}}+1,\ldots ,i_{j_{l}+1}-1\right\}\;,\;l=1,\ldots ,s\;. \end{array}$$

As a concrete example, take $g\in J_{Z}$ uniquely determined by ${g\lceil }_{\left(-\infty ,-2\right]}={\mathrm{id}}_{Z}\lceil_{\left(-\infty ,-2\right]}$ and $\delta_{g}=\left\{-1,0,1,3,4,7\right\}$. Then s=2 as A={3,5}, and consequently $\delta^{\left(1\right)}=\left\{-1,0,1\right\}$, $\delta^{\left(2\right)}=\left\{3,4\right\}$, $\delta^{\left(3\right)}=\left\{7\right\}$, $\lambda^{\left(1\right)}=\left\{2\right\}$, and finally $\lambda^{\left(2\right)}=\left\{5,6\right\}$.

After defining $p_{l}:=|\delta^{\left(l\right)}|\;,r_{l}:=\left|\lambda^{\left(l\right)}\right|$, one achieves the desired decomposition in this case too, by getting

$$\begin{array}{cc} f & =\tau^{m}\theta_{k+1+\sum_{l=1}^{s}\left(p_{l}+r_{l}\right)}^{p_{s+1}}\cdots \theta_{k+1+p_{1}+r_{1}}^{p_{2}}\theta_{k+1}^{p_{1}}\\ \; & =\theta_{m+k+1+\sum_{l=1}^{s}\left(p_{l}+r_{l}\right)}^{p_{s+1}}\cdots \theta_{m+k+1+p_{1}+r_{1}}^{p_{2}}\theta_{m+k+1}^{p_{1}}\tau^{m}\;, \end{array}$$

where the last equality comes again from Equation (5).

Suppose now that

$$f=h_{1}\tau^{m}=h_{2}\tau^{n}\;,\;h_{1},h_{2}\in D_{Z}\;,m,n\in Z\;.$$

Since there exist uniquely determined $k_{i}\in Z$ such that $h_{i}{\lceil_{\left(-\infty ,k_{i}\right]}=\mathrm{id}\lceil }_{\left(-\infty ,k_{i}\right]}$, i=1,2, one firstly gets m=n. Uniqueness of the above decomposition then follows if $h_{1}=h_{2}$. To this goal, we reduce the matter to $h_{1}\neq {\mathrm{id}}_{Z}\neq h_{2}$, the other cases being trivial. Here, since

$$\theta_{l}\theta_{k}=\theta_{k}\theta_{l-1}\;,\;k<l\;,$$

one finds the following “normal order” for $h_{i}$, i=1,2:

$$h_{1}=\theta_{k_{n}}^{p_{n}}\cdots \theta_{k_{2}}^{p_{2}}\theta_{k_{1}}^{p_{1}}\;,\;\;\;\;\;\;\;\;\;\;\;\;\;\;\;\;\;h_{2}=\theta_{r_{m}}^{q_{m}}\cdots \theta_{r_{2}}^{q_{2}}\theta_{r_{1}}^{q_{1}}\;,$$

where m,n∈N, $k_{1},\ldots ,k_{n},r_{1},\ldots ,r_{m}\in Z$, $p_{1},\ldots p_{n},q_{1},\ldots ,q_{m}\in N$, and finally $k_{l+1}>k_{l}+p_{l}$, $r_{s+1}>r_{s}+q_{s}$, for l=1,…,n−1, s=1,…,m−1. This immediately yields $h_{1}=h_{2}$.

Summarising, the map

$$Z{\;}_{\eta }\;\;⋉D_{Z}∋\left(m,h\right)\mapsto h\tau^{m}\in J_{Z}$$

realises an isomorphism between monoids. Therefore, for $f\in J_{Z}$, the unique expression $f=h\tau^{n}$ with $h\in D_{Z}$ and n∈Z, provides the description of $J_{Z}$ as inner semi-direct product between elements of $D_{Z}$ and powers of τ and $\tau^{-1}$.

Concerning the sub-monoid $I_{Z}\subset J_{Z}$, we get

$$\begin{array}{cc} J_{Z}=Z{\;}_{\eta }\;\;⋉D_{Z}= & \left\{h\tau^{n}|h\in D_{Z},\;\;n\in Z\right\}\\ \subset & \left\{h\tau^{n}|h\in I_{Z},\;\;n\in Z\right\}\\ = & Z{\;}_{\eta }\;\;⋉I_{Z}\subset J_{Z}\;, \end{array}$$

and thus we have the equality of monoids

$$Z{\;}_{\eta }\;\;⋉D_{Z}=Z{\;}_{\eta }\;\;⋉I_{Z}=J_{Z}\;.$$

The equality $Z{\;}_{\eta }\;\;⋉E_{Z}=Z{\;}_{\eta }\;\;⋉J_{Z}$ follows analogously. □

Once having established (cf. Remark 4) that spreadability of stochastic processes on the index-set Z can be indifferently investigated by the monoids $I_{Z}$, $J_{Z}$ and $L_{Z}$, in the next section we will see that the intermediate monoid $J_{Z}$ provides a more flexible analysis in the case of the (concrete) boolean $C^{*}$-algebra.

## 5. Spreading Invariant States on the Boolean Algebra

As an application of the previous results, the present section is devoted to the investigation of the spreadability for stochastic processes arising from the so-called boolean commutation relations (7). For such a purpose, the main step will be to show that the monoid $J_{Z}$ acts on the concrete boolean unital $C^{*}$-algebra by unital *-endomorphisms.

Let H be a complex Hilbert space with inner product 〈·,·〉, linear in the first argument. The boolean Fock space $F_{\mathrm{boole}}\left(H\right)$ over H is the direct sum $F_{\mathrm{boole}}\left(H\right)=C⊕H$, and the vacuum vector is $e_{\#}:=1⊕0$. The vacuum vector state is denoted by $\omega_{\#}:=\langle \cdot \;e_{\#},e_{\#}\rangle$.

For γ∈C and f,g∈H, the creation and annihilation operators are defined as follows:

$$b^{†}\left(f\right)\left(\gamma ⊕g\right):=0⊕\gamma f,\;\;\;\;b\left(f\right)\left(\gamma ⊕g\right):={\langle g,f\rangle }_{H}⊕0\;.$$

They are mutually adjoint and bounded.

The concrete boolean $C^{*}$-algebra $b_{H}$ is that generated by all creators and the identity $1\;\;I_{F_{\mathrm{boole}}\left(H\right)}:=I$. Since the *-algebra generated by the $b^{†}$ consists of all finite rank operators, we easily get

(6) $$b_{H}=K\left(C⊕H\right)+CI\;,$$

where K(C⊕H) denotes the $C^{*}$-algebra of compact linear operators on H. Here, we deal with the case $H=\ell^{2}\left(Z\right)$, where the canonical basis is $\left\{e_{j}|j\in Z\right\}$. Therefore

$$\begin{array}{cc} F_{\mathrm{boole}}\left(\ell^{2}\left(Z\right)\right)= & Ce_{\#}⊕\ell^{2}\left(Z\right)=\ell^{2}\left(\left\{\#\right\}⊔Z\right)\;,\\ b_{\ell^{2}\left(Z\right)}= & K(\ell^{2}\left(\left\{\#\right\}⊔Z\right))+CI\;. \end{array}$$

With the notations $b_{j}:=b\left(e_{j}\right)$, $b_{j}^{†}:=b^{†}\left(e_{j}\right)$, we can see that the following boolean commutation relation (in the spirit of [20], pag. 109)

(7) $$b_{i}b_{j}^{†}-\sum_{r,s\in Z}T_{js}^{ir}b_{r}^{†}b_{s}=\delta_{i,j}I$$

holds true with $T_{js}^{ir}=-\delta_{i,j}\delta_{r,s}$, $\delta_{i,j}$ being the usual Kronecker symbol. The above infinite sum is meant (i.e., converges) in the strong operator topology of $B\left(F_{\mathrm{boole}}\left(\ell^{2}\left(Z\right)\right)\right)$, as it was seen in [15], Proposition 3.2.

For the convenience of the reader, we report the following result proved in [10], Proposition 7.1: the unital $C^{*}$-algebra generated by the position operators $\left\{b_{j}+b_{j}^{†}|j\in Z\right\}$ coincides with $b_{\ell^{2}\left(Z\right)}$ and therefore, differently from the analogous one generated by the position operators of the free commutation relations, it acts irreducibly on $\ell^{2}\left(\left\{\#\right\}⊔Z\right)$.

From now on, we use the shorthand notation $Z_{\#}:=\left\{\#\right\}⊔Z$. For each subset $A\subset Z_{\#}$, $P_{A}\in B\left(\ell^{2}\left(Z_{\#}\right)\right)$ will denote the self-adjoint projection onto the closed subspace of $\ell^{2}\left(Z_{\#}\right)$ generated by the $e_{j}$, j∈A.

Thus, $K(\ell^{2}\left(Z_{\#}\right))$ is the $C^{*}$-algebra of compact linear operators acting on $\ell^{2}\left(Z_{\#}\right)$, and for the canonical system of matrix–units $\left\{\varepsilon_{ij}|i,j\in Z_{\#}\right\}$ in $B(\ell^{2}\left(Z_{\#}\right))$, one has

$$b_{j}=\varepsilon_{\#j}\;,\;\;b_{j}^{†}=\varepsilon_{j\#}\;,\;\;b_{i}b_{j}^{†}=\varepsilon_{\#\#}\delta_{i,j}\;,\;\;b_{i}^{†}b_{j}=\varepsilon_{ij}\;,\;i,j\in Z\;.$$

It is well known that the following groups naturally act on $b_{\ell^{2}\left(Z\right)}$ by *-automorphisms (e.g., [4,10,15]):

**-**  the integers Z by all powers of the one-step shift τ and its inverse;
**-**  the group $P_{Z}$ of all permutations moving only finitely many elements of Z.

Such actions are directly implemented by the (2^nd^ quantised action of the) corresponding actions on the canonical basis of $\ell^{2}\left(Z\right)$, that is by Bogolyubov automorphisms (cf. [21]).

In what follows, we show that also $J_{Z}$ acts by unital *-endomorphisms on $b_{\ell^{2}\left(Z\right)}$. Such an action determines the structure of positive normalised functionals which are invariant, that is the spreadable stochastic processes arising from the boolean commutation relations. The reader is referred to [5] for the similar situation involving monotone (and anti-monotone) commutation relations.

Let $K_{o}\left(\ell^{2}\left(Z_{\#}\right)\right)$ be the *-algebra of finite rank operators on the boolean Fock space. On the unital *-algebra $K_{o}\left(\ell^{2}\left(Z_{\#}\right)\right)+CI$, dense in the norm topology in $b_{\ell^{2}\left(Z\right)}$, as for the above mentioned actions of Z and $P_{Z}$, we can define

$$\alpha^{\left(o\right)}:f\in J_{Z}\mapsto \alpha_{f}^{\left(o\right)}\in (K_{o}\left(\ell^{2}\left(Z_{\#}\right)\right){+CI)}^{K_{o}\left(\ell^{2}\left(Z_{\#}\right)\right)+CI}$$

such that

(8) $$\begin{array}{cc} \; & \alpha_{f}^{\left(o\right)}\left(I\right):=I\;,\\ \; & \alpha_{f}^{\left(o\right)}\left(\varepsilon_{kl}\right):=\varepsilon_{f_{\#}\left(k\right)f_{\#}\left(l\right)},\;k,l\in Z_{\#}\;, \end{array}$$

where

$$f_{\#}\left(k\right):=\left\{\begin{array}{cc} f(k) & \mathrm{if}\;k\in Z\;,\\ k & \mathrm{if}\;k=\#\;. \end{array}\right.$$

The $\alpha_{f}^{\left(o\right)}$ are well defined because $\left\{\varepsilon_{kl}|k,l\in Z_{\#}\right\}\subset K_{o}\left(\ell^{2}\left(Z_{\#}\right)\right)$ is a Hamel basis. In accordance to the action of $I_{Z}$ on the monotone $C^{*}$-algebra (cf. [5]), we can prove that $\alpha^{\left(o\right)}$ extends to an action of $J_{Z}$ by unital *-endomorphisms of the boolean $C^{*}$-algebra $b_{\ell^{2}\left(Z\right)}$ by providing an explicit formula (see Theorem 1) for such an action.

On the canonical basis $\left\{e_{j}|j\in Z_{\#}\right\}$ of $\ell^{2}\left(Z_{\#}\right)$, for any $f\in J_{Z}$ we define

$$V_{f}e_{k}:=e_{f_{\#}\left(k\right)}\;,\;k\in Z_{\#}\;,$$

which extends to an isometry on $\ell^{2}\left(Z_{\#}\right)$, denoted again by $V_{f}$. Indeed,

**Lemma** **2.** *For any $f,g\in J_{Z}$, one has*

(9) $$\begin{array}{c} \begin{array}{cc} \; & V_{f}^{*}V_{f}=I\;,\\ \; & V_{f}V_{f}^{*}=P_{\delta_{f}}^{⊥}\;,\\ \; & V_{f}P_{\delta_{g}}V_{f}^{*}=P_{f(\delta_{g})}\;. \end{array} \end{array}$$

**Proof.**  Since the $f\in J_{Z}$ are injective, the $V_{f}$ are isometries. In addition, as for $f\in J_{Z}$ and $k\in Z_{\#}$ we get

$$V_{f}^{*}e_{k}=\left\{\begin{array}{cc} e_{k} & \mathrm{if}\;k=\#\;,\\ e_{f^{-1}\left(k\right)} & \mathrm{if}\;k\in f(Z)\;,\\ 0 & \mathrm{if}\;k\in \delta_{f}\;, \end{array}\right.$$

and therefore the second identity in Equation (9) easily follows.

Since for $g\in J_{Z}$ and $k\in Z_{\#}$, as $f\left(\delta_{g}\right)\subset f\left(Z\right)$, one finds

$$V_{f}P_{\delta_{g}}V_{f}^{*}e_{k}=\left\{\begin{array}{cc} e_{k} & \mathrm{if}\;k\in f(\delta_{g})\;,\\ 0 & \mathrm{otherwise}\;, \end{array}\right.$$

the last identity in Equation (9) is achieved. □

By Equation (6), any $X\in b_{\ell^{2}\left(Z\right)}$ is decomposed as X:=K+γI, where $K\in K(\ell^{2}\left(Z_{\#}\right))$ and γ∈C. Thus, the state at infinity $\omega_{\infty }$ is well defined as

(10) $$\omega_{\infty }\left(X\right):=\gamma \;.$$

**Lemma** **3.** *For $f\in J_{Z}$ and $X\in b_{\ell^{2}\left(Z\right)}$, we get:*

*(i)*  *$V_{f}XV_{f}^{*}\in b_{\ell^{2}\left(Z\right)}$,*
*(ii)*  *$\omega_{\infty }\left(V_{f}XV_{f}^{*}\right)=\omega_{\infty }\left(X\right)$.*

**Proof.**  We start by noticing that $K\left(\ell^{2}\left(Z_{\#}\right)\right)\subset B\left(\ell^{2}\left(Z_{\#}\right)\right)$ is a two-sided ideal, and $f\in J_{Z}$ implies $P_{\delta_{f}}$ is finite rank.

Fix $X=K+\gamma I\in b_{\ell^{2}\left(Z\right)}$. By Equation (9), we get:

(i) $V_{f}\left(K+\gamma I\right)V_{f}^{*}=(V_{f}KV_{f}^{*}-\gamma P_{\delta_{f}})+\gamma I\in b_{\ell^{2}\left(Z\right)}$.
(ii) $\omega_{\infty }\left(V_{f}XV_{f}^{*}\right)=\gamma =\omega_{\infty }\left(X\right)$. □

Our next goal consists in showing it is possible to extend $\alpha^{\left(o\right)}$ in Equation (8) as an action of *-endomorphisms on the whole $C^{*}$-algebra $b_{\ell^{2}\left(Z\right)}$.

For $f\in J_{Z}$, define the linear maps

(11) $$\alpha_{f}\left(X\right)=V_{f}XV_{f}^{*}+\omega_{\infty }\left(X\right)P_{\delta_{f}}\;,\;X\in b_{\ell^{2}\left(Z\right)}\;.$$

**Theorem** **1.** *The map*

$$\alpha :f\in J_{Z}\to \alpha_{f}\in \mathrm{End}(b_{\ell^{2}\left(Z\right)})$$

*given in Equation (11) provides a representation of the monoid $J_{Z}$ in $\mathrm{End}(b_{\ell^{2}\left(Z\right)})$ extending the linear maps given in Equation (8).*

**Proof.**  We start by noticing that the maps in Equation (11) preserve the *-operation, and are unital and bounded.

In addition, for $f\in J_{Z}$ and $X:=K+\gamma I\in K\left(\ell^{2}\left(Z_{\#}\right)\right)+CI=b_{\ell^{2}\left(Z\right)}$, Equations (10) and (9) give

$$\alpha_{f}\left(X\right)=V_{f}XV_{f}^{*}+\omega_{\infty }\left(X\right)P_{\delta_{f}}=V_{f}KV_{f}^{*}+\gamma I\;.$$

For $X_{j}=K_{j}+\gamma_{j}I$, j=1,2, we now check

$$\begin{array}{cc} \alpha_{f}\left(X_{1}X_{2}\right) & =\alpha_{f}\left(K_{1}K_{2}\right)+\gamma_{2}\alpha_{f}\left(K_{1}\right)+\gamma_{1}\alpha_{f}\left(K_{2}\right)+\gamma_{1}\gamma_{2}I\\ \; & =V_{f}K_{1}K_{2}V_{f}^{*}+\gamma_{2}V_{f}K_{1}V_{f}^{*}+\gamma_{1}V_{f}K_{2}V_{f}^{*}+\gamma_{1}\gamma_{2}I\\ \; & =\left(V_{f}K_{1}V_{f}^{*}+\gamma_{1}I\right)\left(V_{f}K_{2}V_{f}^{*}+\gamma_{2}I\right)\\ \; & =\alpha_{f}\left(X\right)\alpha_{f}\left(Y\right)\;, \end{array}$$

and therefore the $\alpha_{f}$ are unital *-endomorphisms of $b_{\ell^{2}\left(Z\right)}$.

Now we check that $f\mapsto \alpha_{f}$ provides an action of $J_{Z}$ on $b_{\ell^{2}\left(Z\right)}$. To this aim, fix $f,g\in J_{Z}$. Exploiting Lemma 3 (ii), Equations (9) and (3), one obtains

$$\begin{array}{cc} \alpha_{f}\alpha_{g}\left(X\right) & =\alpha_{f}\left(V_{g}XV_{g}^{*}+\omega_{\infty }\left(X\right)P_{\delta_{g}}\right)\\ \; & =V_{f}\left(V_{g}XV_{g}^{*}+\omega_{\infty }\left(X\right)P_{\delta_{g}}\right)V_{f}^{*}+\omega_{\infty }\left(V_{g}XV_{g}^{*}+\omega_{\infty }\left(X\right)P_{\delta_{g}}\right)P_{\delta_{f}}\\ \; & =V_{fg}XV_{fg}^{*}+\omega_{\infty }\left(X\right)\left(V_{f}P_{\delta_{g}}V_{f}^{*}+P_{\delta_{f}}\right)\\ \; & =V_{fg}XV_{fg}^{*}+\omega_{\infty }\left(X\right)\left(P_{f(\delta_{g})}+P_{\delta_{f}}\right)\\ \; & =V_{fg}XV_{fg}^{*}+\omega_{\infty }\left(X\right)P_{\delta_{fg}}\\ \; & =\alpha_{fg}\left(X\right)\;. \end{array}$$

Finally, consider $f\in J_{Z}$, and a generic matrix unit $\varepsilon_{ij}$, for $i,j\in Z_{\#}$. For any $e_{k}\in \ell^{2}\left(Z_{\#}\right)$

$$\begin{array}{cc} \alpha_{f}\left(\varepsilon_{ij}\right)e_{k} & =\left\{\begin{array}{cc} \delta_{{}_{j,f^{-1}\left(k\right)}}e_{f_{\#}\left(i\right)} & \mathrm{if}\;k\in f(Z)\cup \{\#\}\;,\\ 0 & \mathrm{if}\;k\in \delta_{f}\;, \end{array}\right. \end{array}$$

and

$$\begin{array}{cc} \alpha_{f}^{\left(o\right)}\left(\varepsilon_{ij}\right)e_{k} & =\left\{\begin{array}{cc} \delta_{{}_{f(j),k}}e_{f_{\#}\left(i\right)} & \mathrm{if}\;k\in f(Z)\cup \{\#\}\;,\\ 0 & \mathrm{if}\;k\in \delta_{f}\;. \end{array}\right. \end{array}$$

Therefore, by linearity $\alpha_{f}\lceil_{K_{o}\left(\ell^{2}\left(Z_{\#}\right)\right)+CI}=\alpha_{f}^{\left(o\right)}$. □

The following result establishes an equivalence between stationary, spreadable and exchangeable processes on the concrete boolean $C^{*}$-algebra, thus realising a version of Ryll–Nardzewski Theorem [8] in our setting.

**Proposition** **5.** *The states on $b_{\ell^{2}\left(Z\right)}$ which are spreading invariant coincide with the stationary and symmetric states:*

$$\begin{array}{cc} S_{J_{Z}}(b_{\ell^{2}\left(Z\right)})= & \left\{\lambda \omega_{\#}+\left(1-\lambda \right)\omega_{\infty }|\lambda \in \left[0,1\right]\right\}\\ = & S_{P_{Z}}(b_{\ell^{2}\left(Z\right)})=S_{Z}(b_{\ell^{2}\left(Z\right)})\;. \end{array}$$

**Proof.**  By Remark 7.4 in [15], one gets

$$S_{Z}\left(b_{\ell^{2}\left(Z\right)}\right)=S_{P_{Z}}\left(b_{\ell^{2}\left(Z\right)}\right)=\left\{\lambda \omega_{\#}+\left(1-\lambda \right)\omega_{\infty }|\lambda \in \left[0,1\right]\right\}\;.$$

Moreover, Remark 4 and Proposition 4 give

$$S_{L_{Z}}\left(b_{\ell^{2}\left(Z\right)}\right)=S_{J_{Z}}\left(b_{\ell^{2}\left(Z\right)}\right)\subseteq S_{Z}\left(b_{\ell^{2}\left(Z\right)}\right)\;.$$

The thesis then follows after showing that

$$\omega_{i}\circ \alpha_{f}=\omega_{i}\;,\;i\in \left\{\#,\infty \right\}\;,\;\;\;f\in J_{Z}\;.$$

Indeed, recall that $e_{\#}$ is invariant for $V_{f}^{*}$, $f\in J_{Z}$. Hence, for any X=K+γI with $K\in K(\ell^{2}\left(Z_{\#}\right))$ and γ∈C, Equation (11), Equations (10) and (9) entail

$$\omega_{\#}\left(\alpha_{f}\left(X\right)\right)=\omega_{\#}\left(V_{f}KV_{f}^{*}+\gamma V_{f}V_{f}^{*}+\gamma P_{\delta_{f}}\right)=\omega_{\#}\left(K+\gamma I\right)=\omega_{\#}\left(X\right)\;.$$

Since for $f\in J_{Z}$, $P_{\delta_{f}}$ is finite rank, Lemma 3 yields

$$\omega_{\infty }\left(\alpha_{f}\left(X\right)\right)=\omega_{\infty }\left(V_{f}XV_{f}^{*}+\omega_{\infty }\left(X\right)P_{\delta_{f}}\right)=\omega_{\infty }\left(X\right)\;.$$

 □

## 6. Conclusions

The investigation of the so-called quantum probability started with the seminal paper [22]. After that, several applications to many fields of mathematics and physics have been established. We mention natural applications to models of quantum statistical mechanics and quantum information, and refer the reader to [3,23] and the literature cited therein for more details, even though the list is very far to be complete, compared with hundreds of interesting papers on the topics. On the other hand, a self-containing treatment of quantum probability, similar to the classical one, is nowhere close to being satisfactory.

An attempt towards a unified version of the probability scheme, including as particular cases both the various models arising from noncommutative realm, and the classical one too, was carried out in [4,10]. There, the concept of quantum stochastic process on a discrete index-set was a main topic of the investigation, and some natural distributional symmetries like stationarity and exchangeability were analysed. We also point out the relevance of quantum stochastic processes on continuous index-sets which was firstly outlined in [24]. Therefore, its development towards a systematic theory could be a very interesting direction for future research.

In order to present some open questions closely related with the present notes, we first recall that, in commutative probability, the extended de Finetti theorem states that sequences of random variables which are either spreadable, or exchangeable, or finally conditionally independent and identically distributed w.r.t. the tail algebra, coincide. In [12], Theorem 1, it was proved that exchangeable boolean stochastic processes are indeed those conditionally independent and identically distributed w.r.t. the tail algebra (known for physical applications as the algebra at infinity, see, e.g., [16]). Consequently, Proposition 5 allows us to achieve the boolean version of the aforementioned theorem. Since similar results were obtained for monotone and *q*-deformed stochastic processes in [5,10], open problems for future investigation could be:

**-**  studying all natural symmetries like stationarity, exchangeability and spreadability, in the case of stochastic processes associated to anomalous commutation relations arising from quantum physics, such as the Fermi case [25], or the more general case of Yang–Baxter–Hecke quantisation [26];
**-**  investigating another prominent example of distributional symmetry, that is the rotatability, for general families of noncommutative random variables, see, e.g., [9,27] for commutative and free cases, respectively.

Coming back to more general problems in quantum probability, one finds direct connections with physics, e.g., in Bose, Fermi, or Boltzamnn particle models. On the other hand, up to our knowledge, there are not yet direct physical applications for exotic commutation relations, such as general *q*-deformations and the other ones satisfying Equation (7) and described in [20]. Among those, we however mention the commutation rules corresponding to the anyonic statistics investigated from a mathematical viewpoint in [28], which could provide promising applications to the physical models described in [29].

Concerning the boolean case treated in the present paper, up to the best knowledge of the authors, the only physical motivations is described in [11] as already pointed out above. There, the first direct relation between the boolean commutation relations and the boolean independence (cf. [30]) has been also established.

Boolean independence might also be related to quantum measurement processes as formulated in [31], and successively in [32] (we acknowledge an anonymous referee for bringing to our attention this possible connection and the interesting reference [32]). Therefore, further open problems for possible future investigation could be:

**-**  providing physical applications of random variables exhibiting exotic commutation rules such as the *q*-relations, q∈(−1,0)∪(0,1) (e.g., [33,34]), and the monotone and anti-monotone ones (e.g., [5,15,30]);
**-**  investigating potential connections between boolean independence and quantum measurement processes, in particular for the models fitted to quantum physics described in [32].
